# Population Genomic Analysis of a Bacterial Plant Pathogen: Novel Insight into the Origin of Pierce's Disease of Grapevine in the U.S.

**DOI:** 10.1371/journal.pone.0015488

**Published:** 2010-11-16

**Authors:** Leonard Nunney, Xiaoli Yuan, Robin Bromley, John Hartung, Mauricio Montero-Astúa, Lisela Moreira, Beatriz Ortiz, Richard Stouthamer

**Affiliations:** 1 Department of Biology, University of California Riverside, Riverside, California, United States of America; 2 Department of Entomology, University of California Riverside, Riverside, California, United States of America; 3 USDA-ARS, Molecular Plant Pathology Laboratory, Beltsville, Maryland, United States of America; 4 Centro de Investigación en Biología Celular y Molecular, Universidad de Costa Rica, San José, Costa Rica; University of Wisconsin-Milwaukee, United States of America

## Abstract

Invasive diseases present an increasing problem worldwide; however, genomic techniques are now available to investigate the timing and geographical origin of such introductions. We employed genomic techniques to demonstrate that the bacterial pathogen causing Pierce's disease of grapevine (PD) is not native to the US as previously assumed, but descended from a single genotype introduced from Central America. PD has posed a serious threat to the US wine industry ever since its first outbreak in Anaheim, California in the 1880s and continues to inhibit grape cultivation in a large area of the country. It is caused by infection of xylem vessels by the bacterium *Xylella fastidiosa* subsp. *fastidiosa*, a genetically distinct subspecies at least 15,000 years old. We present five independent kinds of evidence that strongly support our invasion hypothesis: 1) a genome-wide lack of genetic variability in *X. fastidiosa* subsp. *fastidiosa* found in the US, consistent with a recent common ancestor; 2) evidence for historical allopatry of the North American subspecies *X. fastidiosa* subsp. *multiplex* and *X. fastidiosa* subsp. *fastidiosa*; 3) evidence that *X. fastidiosa* subsp. *fastidiosa* evolved in a more tropical climate than *X. fastidiosa* subsp. *multiplex*; 4) much greater genetic variability in the proposed source population in Central America, variation within which the US genotypes are phylogenetically nested; and 5) the circumstantial evidence of importation of known hosts (coffee plants) from Central America directly into southern California just prior to the first known outbreak of the disease. The lack of genetic variation in *X. fastidiosa* subsp. *fastidiosa* in the US suggests that preventing additional introductions is important since new genetic variation may undermine PD control measures, or may lead to infection of other crop plants through the creation of novel genotypes via inter-subspecific recombination. In general, geographically mixing of previously isolated subspecies should be avoided.

## Introduction

Invasive plant pathogens have had a substantial effect on agriculture and native plant communities within the US. The economic costs of these invaders are substantial [1]. Two infamous examples of successful fungal invaders include the agents of Dutch elm disease (*Ophiostoma ulmi*) and chestnut blight (*Cryphonectria parasitica*). Sometimes the disease risk is recognized prior to invasion, so strategies can be adopted to prevent its importation, as in the case of the bacteria *Ralstonia solanacearum* race 3 biovar 2 (a threat to potatoes) and of *Xanthomonas oryzae* (a threat to rice). However, a problem that is rarely considered is how best to deal with an invasive bacterium once it is established. Here we consider a case of a bacterium present in the United States for at least 130 years. Our primary goal has been to establish that it is indeed an introduced pathogen; but in doing we find that, even after all this time, there are still good reasons to prevent additional introductions.

Pierce's disease of grape is caused by infection of the xylem vessels by the bacterium *Xylella fastidiosa*. Initial symptoms include leaf scorch and stunted growth, usually followed by vine death in 1–5 years. The disease was first described by Newton Pierce following the infection of some 40,000 acres of grapes in the 1880s in Anaheim, Orange Co., California [2]. Stoner [3] has suggested that the decline of the fledgling grape industry in Florida the 1890s was due to the same disease. However, European grapes were first planted in California around 1770 in the Missions and apparently grew well [2], suggesting that the disease was introduced into California later, and Hewitt [4] proposed that it originated in the Gulf coastal plains of the US, given the resistance of the wild grape species found there. For this reason, researchers have implicitly assumed that Pierce's-disease causing *X. fastidiosa* were native to the US.

**Figure 1:**
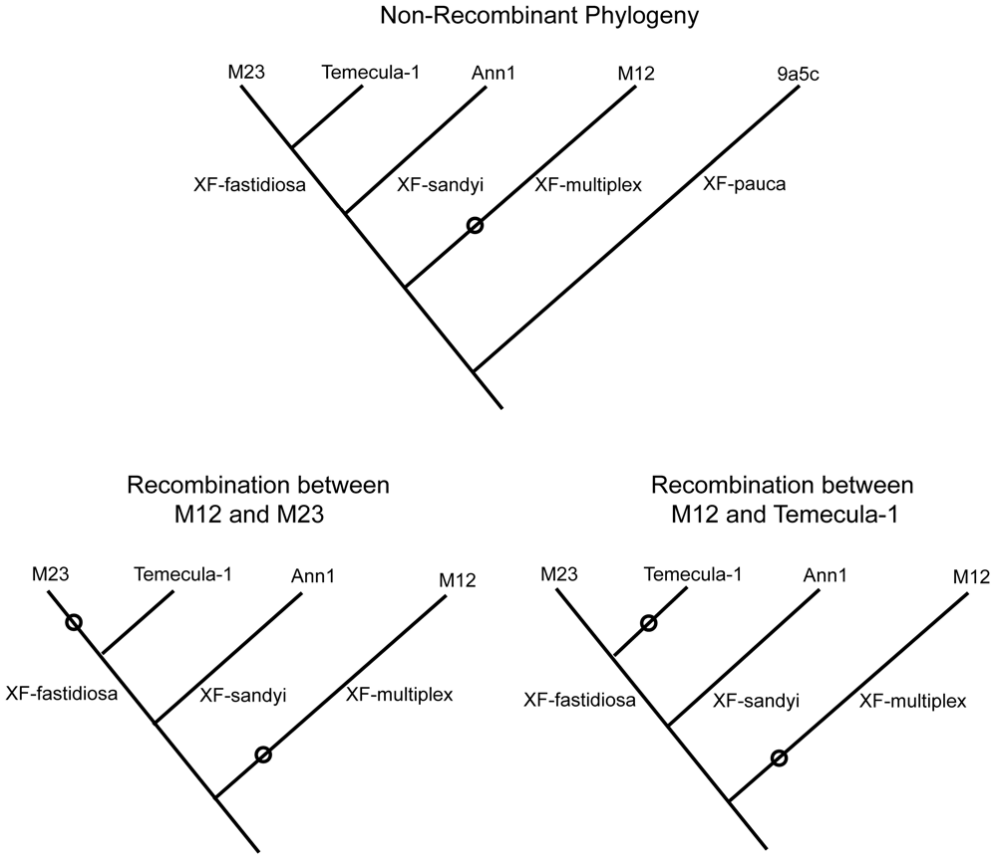
The phylogeny of the major groups of *X. fastidiosa* showing the patterns that can detect recombination of *X. fastidiosa* subsp. *multiplex* DNA into *X. fastidiosa* subsp. *fastidiosa*. The top figure shows the relationships of the four subspecific clades (from Schuenzel et al. 2005) labeled with the sequenced genomes: M23 and Temecula-1 (*X. fastidiosa* subsp. *fastidiosa*), Ann1 (*X. fastidiosa* subsp. *sandyi*), and M12 (*X. fastidiosa* subsp. *multiplex*) from the US, and 9a5c (*X. fastidiosa* subsp. *pauca*) from Brazil. To track recombination, a unique SNP is shown (O) in the *X. fastidiosa* subsp. *multiplex* branch. The lower trees show how recombinational transfer of *X. fastidiosa* subsp. *multiplex* DNA to one of the *X. fastidiosa* subsp. *fastidiosa* forms leaves a characteristic pattern with the non-recombining *X. fastidiosa* subsp. *fastidiosa* remaining identical to *X. fastidiosa* subsp. *sandyi*. Note that the same patterns would be created if recombination involved the transfer of a unique SNP from *X. fastidiosa* subsp. *sandyi*. Branch lengths are not scaled to divergence.

*X. fastidiosa* is a member of the gamma proteobacteria. It is restricted to the Americas and, in addition to Pierce's disease, causes a wide range of leaf-scorch and other diseases in commercially important plants in the Americas including almond leaf scorch, phony peach disease, plum leaf scald, olive leaf scald, citrus variegated chlorosis (CVC), and coffee leaf scorch (CLS) [5]. *X. fastidiosa* is genetically diverse and four subspecific groupings have been suggested [6], [7]: subsp. *pauca* causing CVC and CLS in South America; and, in North America, subsp. *sandyi* causing oleander leaf scorch, subsp. *multiplex* causing diseases in many agricultural and commercial trees including almond, peach, maple, sycamore, and oak, and subsp. *fastidiosa* causing Pierce's disease of grape. Their evolutionary relationships are shown in the upper diagram in Figure 1 [based on Schuenzel et al [7]]. Although these genetically distinct subspecies generally infect different plant hosts, this is not always the case. For example, both *X. fastidiosa* subspp. *multiplex* and *fastidiosa* infect almond [8].

*X. fastidiosa* is dependent for its transmission upon xylem-feeding insects (primarily leafhoppers). Dependence upon an insect vector is likely to be important in restricting the spread of this pathogen across geographical and host barriers. Direct plant-to-plant infection is precluded (except by grafting), so even if an infected plant is imported into a disease-free area, the insect vector must feed on infected plants and transfer it to an appropriate new host for the disease to spread. There has only been a single reported case of Pierce's disease in Europe [9]. This was apparently in a grapevine imported from the US, but the disease failed to spread. The limitation imposed on transmission dynamics by the vector has been dramatically illustrated following the recent invasion of Southern California by the glassy-winged sharpshooter (*Homalodisca vitripennis*). The introduction of this new vector, with a broad ecology and good dispersal ability that contrasted with the native vectors, resulted in a rapid increase in Pierce's disease that severely affected wine growing in the region [see Redak et al. [10]].

The belief that the causal agent of Pierce's disease was native to the Gulf coastal plains or some other part of the US and introduced to southern California in the late 1800s, although compelling, is not supported by population genetic data. Extensive genetic evidence has shown that Pierce's disease is caused by a single related group of *X. fastidiosa* genotypes [11]–[13] identified as the *X. fastidiosa* subsp. *fastidiosa* subspecific form [6], [7], [14]–[16]. Schuenzel et al. [7] noted a lack of genetic diversity in this subspecies based on the DNA sequence of 10 genes from 9 isolates (7 from grape, 2 from almond; 8 from California, 1 from Florida). Consistent with the original hypothesis, they suggested this lack of genetic variation might be due to strong selection imposed by grape and/or almond; however, Yuan et al. [16] tested both the geographical Gulf-origin hypothesis and the host-induced selection hypothesis by investigating if the level of genetic diversity was higher outside of California and/or on alternate plant hosts. They used multi-locus sequence typing (MLST) [17] based on sequencing 4161 bp derived from 7 housekeeping genes, and typed 86 *X. fastidiosa* subsp. *fastidiosa* isolates. There was no increase in overall genetic diversity even though samples from 13 additional host plants were included, and samples came from six different US states including California, Texas, and Florida. Indeed, there was no sign of any evidence of significant geographical variation or genetic differences among isolates from different plant hosts.

These data suggested an alternative hypothesis: that a single genotype of the Pierce's disease causing *X. fastidiosa* subsp. *fastidiosa* was introduced into the US some time shortly before the Anaheim outbreak of the 1880s. This invasion hypothesis raises two immediate questions. First, are the genetic data consistent with all N. American isolates evolving from a single introduced genotype? Second, where is the geographical source of the introduction?

## Results and Discussion

### 1. Are All N. American *X. fastidiosa* subsp. *fastidiosa* Derived from a Single Genotype?

The MLST analysis of 86 isolates from across the U.S. [16] identified 4 sequence types (STs), with 86% of the isolates defined identically as ST1. Adding ST2, the second most frequent sequence type (8 isolates), and ST3 (1 isolate), both of which differed from ST1 by a single base pair, accounted for 97% of the isolates (see Table 1). This pattern is clearly consistent with recent common ancestry; however ST4 (3 isolates) does not, at first sight, seem to fit with this hypothesis. Although ST4 was found to be identical to ST1 across 6 of the 7 genes, in the seventh gene (*cysG*), it differed at 11 sites. However, all of these 11 changes corresponded to differences between *X. fastidiosa* subsp. *fastidiosa* and another North American subspecies *X. fastidiosa* subsp. *multiplex*, indicating that the variation was generated by homologous recombination between the subspecies [16]. The ST4 *cysG* allele differs from the most frequent allele in subsp. *multiplex* at two sites, but these are unlikely to be post-recombinational mutations since both sites are polymorphic for the relevant nucleotides within subsp. *multiplex*. Other examples of recombination have been identified in *X. fastidiosa* [16], [18], [19]. These observations are important because they suggest that the coding genes of *X. fastidiosa* subsp. *fastidiosa* could diversify from a single ancestor in at least two ways: via point mutation and via recombination with another subspecific type. Additionally, we can expect genetic diversification among initially identical genomes due to the high mutation rate at microsatellite sites, due to internal recombination, duplication, or deletion involving dispersed genomic repeats (such as similar, but non-identical, viral elements), and due to the accumulation of novel viral sequences.

**Table 1:** Multi-Locus Sequence Typing (MLST) of 24 isolates from Costa Rica, together with data from 86 US isolates of *X. fastidiosa* subsp. *fastidiosa*, 21 US isolates of *X. fastidiosa* subsp. *sandyi*, plus representative data from *X. fastidiosa* subsp. *multiplex* and *X. fastidiosa* subsp. *pauca*.

		MLST genes listing allele numbers^1^								
Sequence Type	Number isolates	leuA	petC	malF	cysG	holC	nuoL	gltT	Isolate example	Genome alias
***X. fastidiosa*** subsp. ***fastidiosa*** **: Costa Rica**										
ST17	5	1	1	10	12	18	10^2^	1	COF0223	
ST55	1	1	1	10	12	18	10^2^	10	COF0406	
ST57	1	1	1	10	12	18	11^2^	10	COF0405	
ST20	3	1	1	10	12	17	11^2^	11	COF0222	
ST21	2	10	1	10	14	15	11^2^	12	COF0245	
ST19	1	10	1	10	14	15	11^2^	1	COF0209	
ST52	1	10	1	10	14	18	10^2^	1	COF0402	
ST47	4	13	1	10	23	20	5	1	COF0396	
ST18	1	9	1	9	13	14	5	10	PD0212	
ST56	1	11	9	11	15	17	12^2^	10	COF0404	
ST33	3	11	9	14	15	19	13	10	COF0394	
ST54	1	11	9	11	25	19	12^2^	1	COF0412	
***X. fastidiosa*** subsp. ***fastidiosa*** **: US** 3										
ST1	74	1	1	1	1	1	1	1	PD0001ALS0300	Temecula-1 M23
ST2	8	1	1	4	1	1	1	1	PD0016	
ST3	1	1	1	1	20	1	1	1	LUP0215	
ST4	3	1	1	1	4^4^	1	1	1	PD0014	
***X. fastidiosa*** subsp. ***sandyi*** **: US** 3										
ST5	21	2	2	2	2	2	2	2	OLS0002	Ann1
***X. fastidiosa*** subsp. ***multiplex*** **: US** 3										
ST6	-	3	3	3	3	3	3	3	ALS0003	Dixon
ST7	-	3	3	3	7	3	3	3	ALS0299	M12
ST9	-	3	3	5	5	4	3	4	OAK0017	
ST10	-	5	4	3	3	6	3	5	PP0027	
ST26	-	5	3	3	3	6	3	5	PLP0070	
ST39	-	3	3	5	19	4	3	7	LIQ0090	
***X. fastidiosa*** subsp. ***pauca*** **: Brazil** 3										
ST11	-	7	7	7	9	10	8	8	CVC0145	
ST13	-	7	6	7	9	10	7	8	CVC0018	9a5c
ST14	-	8	8	8	11	12	9	9	COF0239	
ST16	-	7	6	8	10	11	8	8	COF0238	

1 For details of the MLST scheme see Yuan et al. [16].

2 Alleles with a 30 bp deletion.

3 Data from Yuan et al. [16], except isolate LIQ0090 (ST39) (see Methods).

**Figure 2:**
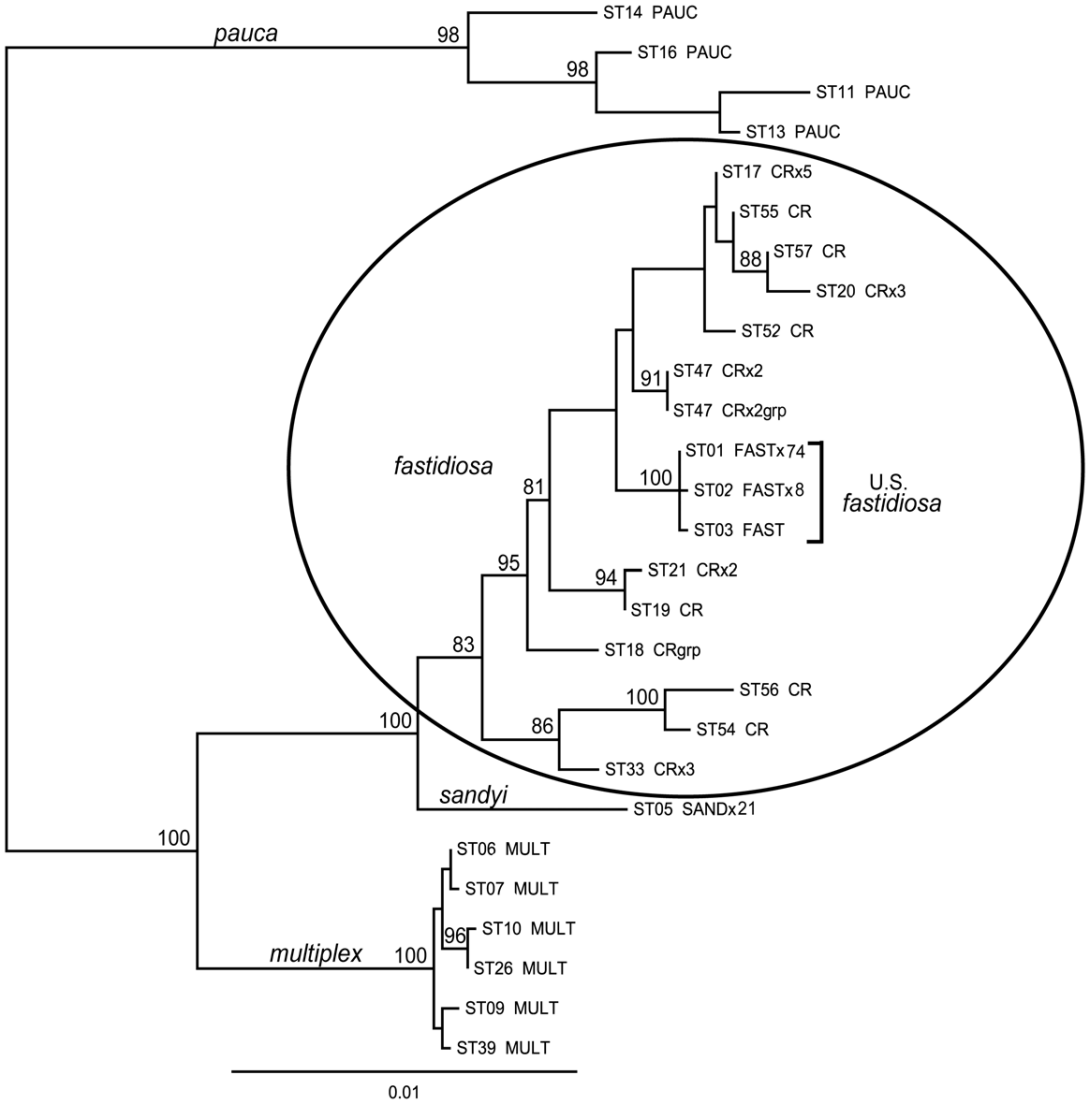
Maximum likelihood phylogeny of *X. fastidiosa* showing U.S. *X. fastidiosa* subsp. *fastidiosa* sequence types (STs) nested within the Costa Rican STs. The circle encompasses all *X. fastidiosa* subsp. *fastidiosa* STs. The other subspecies are named on their ancestral branch. All unique STs are shown from 83 U.S. and 24 Costa Rican (CR) samples of subsp. *fastidiosa* and 21 US samples of subsp. *sandyi*. The number of isolates/ST is shown by xN. All CR isolates were from coffee except 3 from grape (designated by “grp”). *X. fastidiosa* subspp. *multiplex* and *pauca* are represented by a sample of sequence types (see Table 1). All bootstrap values >80% are shown and the scale bar defines 1% sequence divergence.

4 Recombinant allele, hence ST4 was not included in Figure 2.

The listed isolates were used to create Figure 2.

The MLST data showed a level of polymorphism consistent with evolution from a single recent common ancestor, given the additional input of discrete blocks of variation due to recombination (e.g. the cysG allele of ST4). This conclusion is based on results from a diverse array of *X. fastidiosa* subsp. *fastidiosa* isolates; however the analysis involved just 7 genes. The invasion hypothesis predicts that a comparison of whole genomes of *X. fastidiosa* subsp. *fastidiosa* (with an estimated 2066 genes; Van Sluys et al. [20]) should show the same pattern of very few point mutation differences. This prediction was tested using the two available sequenced genomes of *X. fastidiosa* subsp. *fastidiosa*: Temecula-1 isolated from grape in 1998 [20]; and M23 isolated from almond in 2003 [21]. We also used information from the M12 genome [21], which is *X. fastidiosa* subsp. *multiplex*.

If the Temecula-1 and M23 isolates had evolved from a single common ancestor in less than about 150 years, then our prediction was that all genomic differences between these 2.5 Mb genomes could be explained by a limited number of genetic changes: (a) point mutations; (b) small scale length changes at microsatellite (simple sequence repeat) sites; (c) small indels (<20 bp); (d) inter-subspecific recombination; (e) internal genomic processes such as recombination across dispersed non-identical duplicate regions, and (f) larger indels.

Our first goal was to identify differences defining fairly large-scale genomic events corresponding to changes of type (d), (e), and (f). The analysis identified 29 such regions, consisting of 5 indels (>400 bp), totaling 17.8 kb, plus 24 “variable” regions where the local sequence divergence between the two genomes was greater than 1% (Table 2). These variable regions accounted for only 2% of the genome (51.5 kb).

**Table 2:** Comparison of Temecula-1 and M23 *X. fastidiosa* genomes.

Region		Number	Average Length	% of genome
Constant		23	107317	97.96
	SNPs	12	1 per 205690	
	Microsatellites (SSRs)	27	1 per 91418	
	Small Indels (<20 bp)	5	1 per 493656	
Variable - All		24	2146	2.04
	Recombination-M23^1^	6	3060	0.73
	Recombination-Tem^1^	2	1280	0.10
	Duplicate Associated	10	1679	0.67
	Other	6	2465	0.54
Indels (>400 bp)		5	3567	

1 see Table 3.

“Variable” regions of the aligned genomes were regions with >1% sequence divergence (see Methods). All other regions were classified as “Constant”. Variable regions were further classified into four subgroups: Recombination-M23 and Recombination-Tem (evidence of recombination of *X. fastidiosa* subsp. *multiplex* into M23 and Temecula-1 respectively); Duplicate Associated (at least one other copy of the region present in the genomes); and Other (unique variable sequence of unknown origin). Large Indels (>400 bp) were also recorded.

Within the variable 2%, there were 8 regions (averaging 2.6 kb in length) of apparent recombination with *X. fastidiosa* subsp. *multiplex* (Table 3), based on a statistically significant (p <0.001) sequence match to the M12 *X. fastidiosa* subsp. *multiplex* genome. Specifically, inter-subspecific recombination was established if a large directional excess of informative sites was observed. An informative site is one that is not consistent with the overall phylogeny, as diagrammed in the two lower trees in Figure 1. If the two *X. fastidiosa* subsp. *fastidiosa* sequences differed, with one genome (called #1) having the same nucleotide as *X. fastidiosa* subsp. *sandyi* (consistent with the phylogeny) whereas the other genome (#2) having a nucleotide identical to *X. fastidiosa* subsp. *multiplex*, which is inconsistent with the phylogeny, then recombination is indicated, although the occurrence of a convergent mutation (i.e. homoplasy) is also possible. If there are many such sites clustered in a region of DNA that indicate the same recipient (i.e. genome #2 is consistently the same, either Temecula-1 or M23), then recombination is confirmed (Table 3).

**Table 3:** Evidence for homologous recombination of *X. fastidiosa* subsp. *multiplex* DNA (based on the M12 genome) into *X. fastidiosa* subsp. *fastidiosa* genomes M23 and Temecula-1 (Tem).

		Informative sites^1^				Location (Temecula-1)	
Recipientgenome	Length	M12 same as M23	M12 same as Tem	Chi-Square(1df)^2^	Named genes^3^	Start	End
M23	1682	25	0	25.0	-	19651	21332
M23	2122	12	0	12.0	uvrD	47370	49491
M23	5174	52	2	46.3	pyrE	154801	159974
M23	4616	109	0	109.0	pspB	391781	396396
M23	3932	20	1	17.2	rpmB&G,cls, gst	583453	587384
Temecula-1	1425	3	39	30.9	-	1168474	1169898
Temecula-1	1134	0	72	72.0	-	1173029	1174162
M23	837	22	3	14.4	-	1174467	1175303

1 Informative sites are where M23 and Tem differ with M12 = M23 and Tem = Ann1 or M12 = Tem and M23 = Ann1 as shown in figure 1.

2 ^χ2^>10.8 corresponds to p<0.001.

3 Does not include hypothetical genes or those identified only by possible function.

The remaining “constant” regions made up the vast majority (98%) of the genome (Table 2). While the variable regions had, by definition, a high density of genetic differences, the constant regions (totaling 2,468,281 bp) contained very little. There were 5 small indels, and the expected microsatellite site variation (27 sites; Table 2). Yuan et al. [16] observed that of 22 microsatellite loci identified by Lin et al. [13], 15 differed between Temecula-1 and M23. Microsatellite sites diverge rapidly due to their high mutation rate; however, point mutations accumulate more slowly. Consistent with this expectation, only 12 SNPs were found, giving an average frequency of 1 SNP per 205,690 bp, i.e. 0.0005% site polymorphism. This extraordinarily low level of genetic difference suggests very recent common ancestry. Given that Temecula-1 and M23 were isolated at different times (5 years apart), in different places (approximately 200 miles apart), and on different hosts (grape vs. almond) this result provides additional strong support to the hypothesis that *X. fastidiosa* subsp. *fastidiosa* was introduced into the US as a single genotype.

### 2. The Recombination Paradox and Allopatry

Yet another source of support for the invasion hypothesis is provided by the clear phylogenetic signal that distinguishes the three N. American *X. fastidiosa* subspecies, consistent with common ancestry more than 15,000 years ago [7]. The pattern of subspecific relationships, shown diagrammatically in upper Figure 1, has remained robust (with 100% bootstrap support) to the addition of more isolates (see 16). This pattern indicates that over the last 15,000 years genetic exchange between the subspecies has been rare, since recombination creates a reticulate pattern of evolution that limits the development of distinct clades. However, the MLST surveys of *X. fastidiosa* revealed evidence of inter-subspecific recombination [16], [18]. Furthermore, our comparison of the Temecula-1 and M23 genomes indicated at least 8 examples of recombination with *X. fastidiosa* subsp. *multiplex* (Table 3) that occurred over the short period of time since the two genomes shared a common ancestor (based on the similarity of the “constant” regions). This paradox of an apparent absence of historical recombination despite evidence of recent recombination is resolved if the subspecies evolved in allopatry (i.e. geographically separated) and have only come into sympatry relatively recently. In the case of the *X. fastidiosa* subsp. *fastidiosa* in the US, under the invasion hypothesis, sympatry would have been initiated when it was first introduced.

### 3. Tropical vs. Temperate Rates of Evolution?

Another interesting feature of the phylogenetic relationships of the N. American subspecies is that since the evolutionary split of *X. fastidiosa* subsp. *multiplex* from *X. fastidiosa* subspp. *fastidiosa* and *sandyi*, the rate of evolution in *X. fastidiosa* subsp. *multiplex* has been substantially slower. Schuenzel et al. [7] used the rate of synonymous substitutions in these subspecific branches to estimate divergence times, and found that the rate in the *X. fastidiosa* subspp. *fastidiosa*/*sandyi* branch was more than 2.6 times faster (leading to estimated divergence times of roughly 44.5K yrs using the *X. fastidiosa* subspp. *fastidiosa*/*sandyi* branch vs. 17K yrs using the *X. fastidiosa* subsp. *multiplex* branch). Assuming that synonymous substitutions are generally neutral or nearly so and that the per division mutation rate is constant across the subspecies, then, based on neutral theory, the cause of this difference must be the number of cell divisions per year. This difference can be explained if *X. fastidiosa* subsp. *multiplex* has evolved in a more temperate environment, with its shorter growing season, compared to the other two subspecies. Such an explanation is consistent with *X. fastidiosa* subsp. *fastidiosa* evolving further south, while *X. fastidiosa* subsp. *multiplex* evolved in North America. The view that *X. fastidiosa* subsp. *multiplex* is native to North America is further supported by the observation of Yuan et al. (2010) that, even based on a small sample of *X. fastidiosa* subsp. *multiplex*, it showed a level of nucleotide polymorphism about five times higher than *X. fastidiosa* subsp. *fastidiosa*.

### 4. Geographical Origins of *X. fastidiosa* subsp. *fastidiosa*

The invasion hypothesis requires an answer to the crucial question: where did *X. fastidiosa* subsp. *fastidiosa* originate? Hewitt's [4] hypothesis that it was native to the US Gulf states is unsupported: of 11 isolates from Texas, 10 were of ST1, one of ST2, a distribution typical of other areas (see 16). Thus the Texas isolates, like those from other parts of the US, lack the genetic diversity that would be expected if *X. fastidiosa* subsp. *fastidiosa* had a long history in the area.

*X. fastidiosa* is restricted to the Americas. In South America, *X. fastidiosa* subsp. *pauca* has been extensively studied since its identification as the cause of CVC [22], including early genomic sequencing [23]. However, *X. fastidiosa* subsp. *pauca* is highly differentiated from the other three subspecies (about 3%; see 7), suggesting long-term isolation, which makes South America an unlikely source for *X. fastidiosa* subsp. *fastidiosa*. A more probable source is Central America. It has been found in Costa Rica [24], and is thought to occur throughout the Americas (Montero-Astúa et al 2008), but most importantly Montero-Astúa et al. [25], [26] noted that Costa Rican isolates were more genetically similar to isolates from the US than to those from Brazil. To test the possibility that the subspecies *X. fastidiosa* subsp. *fastidiosa* had its origins in Central America, we classified 24 Costa Rican isolates from coffee, *Coffea arabica* (21 isolates) and grape, *Vitis vinifera* (3 isolates) using our MLST scheme (Table 1; data available at http://www.pubmlst.org/xfastidiosa).

The Costa Rican isolates defined 12 sequence types (STs). The similarity between these isolates and the *X. fastidiosa* subsp. *fastidiosa* isolates from the US was immediately apparent: 25% of the 168 gene copies scored (7 loci x 24 isolates) were alleles that had previously been observed in all *X. fastidiosa* subsp. *fastidiosa* from the US, whereas none had been previously observed in *X. fastidiosa* subsp. *multiplex* or *X. fastidiosa* subsp. *sandyi* isolates from the US, or in *X. fastidiosa* subsp. *pauca* from Brazil (Table 2; see also 16).

To establish this relationship more precisely, we used a phylogenetic approach. Overall we have typed approximately 350 isolates of *X. fastidiosa* (unpublished data); however, for the tree shown (figure 2), we included the 24 Costa Rican isolates, data from Yuan et al. [16] on *X. fastidiosa* subspp. *fastidiosa* and *sandyi*, plus representative sequence types of *X. fastidiosa* subsp. *multiplex* and *X. fastidiosa* subsp. *pauca* (Table 1). The maximum likelihood analysis used a concatenation of the DNA sequence from the 7 MLST genes to ensure a broad sampling of the genome.

The phylogenetic tree (Figure 2) shows the subspecific groupings with all of the Costa Rican (CR) isolates within the *X. fastidiosa* subsp. *fastidiosa* subspecific clade. Moreover, these CR isolates showed much greater variability than the US representatives of the subspecies, which corresponded to a tiny subset nested within the *X. fastidiosa* subsp. *fastidiosa* variation. This level of variation represents many thousands of years of diversification, based on the dating of the *X. fastidiosa* subspp. *fastidiosa*/*sandyi* split at more than 15,000 years ago [7].

Montero-Astúa et al. [26] previously noted that *X. fastidiosa* is probably native to Costa Rica, and the genetic data, showing a wide range of genotypes isolated from coffee, indicate that *X. fastidiosa* subsp. *fastidiosa* has probably been infecting coffee plants for some time. However, coffee has been successfully grown since its introduction in the 18^th^ century and infection by *X. fastidiosa* was only recently confirmed [27], suggesting *X. fastidiosa* infection of coffee has historically caused relatively mild symptoms. In contrast, Goheen et al. [24] confirmed *X. fastidiosa* in grape in 1979 and attributed the long-term failure of the Costa Rican grape industry to Pierce's disease.

### 5. An Invasion Hypothesis

A final consideration concerns how *X. fastidiosa* subsp. *fastidiosa* may have arrived in the US. Many different crops were imported into California in the period from 1850-1870 and in 1863 the state of California tried to stimulate the production of new crops through a bounty law. Bounties were awarded for many different crops, and the first grower to produce 10 chests of coffee was to receive a bounty of $1,000 [30]. The focal point of the Pierce's Disease outbreak was Anaheim where large tracts of land had been converted to vineyards and the disease spread from there to the surrounding counties [2]. No other grape growing areas either to the north or to the south were affected by the disease until later, suggesting that an infected plant was imported directly into the Anaheim area. Since we have established that coffee plants carry *X. fastidiosa* subsp. *fastidiosa* in Costa Rica, and that a single sequence type (ST47) was able to infect both grape and coffee, we looked for evidence of the importation of coffee plants into Southern California around that time, and found that coffee plants were indeed imported from Central America (Nicaragua) and sold through a nursery in Los Angeles as early as 1855 [31]. Of course, the bacterium could have been introduced elsewhere in the U.S. (such as Texas or Florida which have long had problems growing grapevines) and spread to Anaheim, and it could have been introduced in a plant other than coffee; however, the juxtaposition of the first documented outbreak of Pierce's disease at a time and place that coffee plants were being imported from Central America certainly makes a compelling albeit circumstantial case.

### 6. The Origins of *X. fastidiosa* subsp. *sandyi*

The explanation given earlier of the relatively slow evolutionary rate of *X. fastidiosa* subsp. *multiplex* relative to *X. fastidiosa* subsp. *fastidiosa* and *X. fastidiosa* subsp. *sandyi*, suggests that *X. fastidiosa* subsp. *sandyi* also has its origins in a more tropical region. *X. fastidiosa* subsp. *sandyi* causes oleander leaf scorch, which was first detected in Southern California in the 1980s. The 21 isolates (from California and Texas) so far examined (Yuan et al. 2010) were all of a single sequence type (ST5) (see Table 1). These pieces of information taken together strongly suggest that there was a single introduction of *X. fastidiosa* subsp. *sandyi* into the US about 30 years ago; however, as yet we have no direct information regarding the geographical source of the introduction.

### 7. Conclusions

The phylogenetic pattern of *X. fastidiosa* subsp. *fastidiosa*, with the high diversity of Costa Rican isolates contrasting with the homogeneity of the North American isolates (Figure 2), supports the hypothesis that *X. fastidiosa* subsp. *fastidiosa* was introduced into the United States from Central America. In addition, the results show that a range of genotypes can infect grape. ST18 was found in a single grape isolate, and ST47 was isolated from both grape and coffee. These two sequence types differ from the US grape isolates by a minimum of 23 bp (0.55%) and 12 bp (0.29%), respectively, providing further evidence that selection for success in grape is unlikely to have driven the observed lack of variation in North American isolates.

The finding of extensive Central American variation in *X. fastidiosa* subsp. f*astidiosa* and of evidence of inter-subspecific recombination show that *X. fastidiosa* subsp. *fastidiosa* within the US is far from being evolutionarily stable. Novel genetic variation is already accumulating via recombination, and the possibility of the accidental introduction of new genotypes from Central America represents a real risk. This last point is important. Even though PD-causing *X. fastidiosa* has been established in the US for more than 100 years, it remains important to prevent additional introductions of this subspecies. The effect of increased genetic diversity is likely to be two-fold. First, the nature of PD may change. Specifically, the effectiveness of control methods may deteriorate. This is a particular concern for grape varieties bred for resistance to the more-or-less homogeneous bacterial genotype that currently prevails. Second, we anticipate that as *X. fastidiosa* subsp. *fastidiosa* diversifies genetically it is very likely to invade novel plant hosts. *X. fastidiosa* is known to infect a very wide range of plant hosts [28] and the genetic novelty created by the progressive mixing of two subspecies creates a significant opportunity for adaptation to new hosts. Hybridizations of this kind among fungal plant pathogens, both intra- and inter-specific, have created major problems [29], including rapid evolutionary change on the same host (e.g. between the North American and European forms of the casual agent of Dutch elm disease, *Ophostoma novo-ulmi*), and host shifts (e.g. a hybrid *Phytophthera* in Europe that infects alder, a host that neither parental species can infect). The mixing of *Xylella* subspecies not only poses a problem for North America. The other parts of its range, i.e. Central and South America, also have their own *X. fastidiosa* subspecies suggesting that in general precautions should be taken against importing non-native subspecies of this pathogen throughout the Americas.

We have demonstrated that the nationwide and economically damaging establishment of Pierce's disease is almost certainly the result of the introduction of a single infected plant. The same is likely to be true of recent arrival of oleander leaf scorch, caused by *X. fastidiosa* subsp. *sandyi*. These observations make two opposing points. First, as noted earlier, it supports the view that vector-transmitted plant pathogens such as *X. fastidiosa* rarely invade across geographical barriers otherwise we would not be detecting such unique invasions. Second, we need to recognize that the movement of live plant material by the nursery industry, or potentially the cut-flower industry, is not risk free. This is important to bear in mind given that *X. fastidiosa* subsp. *pauca* is one of the few plant pathogens on the USDA list of plant protection and quarantine select agents (http://www.selectagents.gov). This is the form that causes CVC and CLS in Brazil; however, we do not know what native plants may carry this pathogen and what, if any, symptoms they may show. Thus the risk of introduction persists.

In summary, we have provided five independent kinds of evidence that strongly support our invasion hypothesis: a lack of genetic variability in *X. fastidiosa* subsp. *fastidiosa* found in the US, a pattern that was supported by a whole genome analysis and consistent with all US isolates sharing recent common ancestry; evidence for historical allopatry of the subspecific types based on the finding of recent but not older inter-subspecific recombination; evidence that *X. fastidiosa* subsp. *fastidiosa* evolved in a more tropical climate than the apparently native North American subspecies *X. fastidiosa* subsp. *multiplex* based on their rates of evolution at synonymous sites; much greater genetic variability in the potential source population of Central America, variation within which the US genotypes are nested; and, finally, evidence of importation of coffee plants, a carrier of *X. fastidiosa* subsp. *fastidiosa*, from Central America directly into southern California just prior to the first known outbreak of the disease. Thus contrary to the long-standing assumption that Pierce's disease is native to the US (see 4), there is overwhelming evidence that this disease was introduced into the United States from Central America as a single isolate, perhaps being imported in an infected coffee plant directly to Southern California in the 1860s.

## Methods

### Bacterial Isolates Used

Details of the 15 Costa Rican isolates are provided in Table 4. Information on the other isolates used in the analysis are given in Yuan et al. [16], except for LIQ0090, an additional isolate that was extracted from *Liquidambar styraciflua* in Riverside, Ca in 2009, CVC0145, alias ICPB 50031 [5], and COF0238, alias Funde-2 [26]. The gene sequences of the new MLST alleles found in this study are available at GenBank under the following accession numbers: FJ610156-60, FJ610162-9, FJ610171-6, FJ610178-85, FJ610187, FJ610189-90, FJ610192-98, FJ610200-7, FJ610209-11, FJ610213-22, FJ965544, FJ965546, HM243595-615 where hyphens denote ranges (all other allele sequences are listed in ref. 16), plus all of the sequence data and isolate details are available at www.pubMLST.org/xfastidiosa.

**Table 4:** *Xylella fastidiosa* isolates from Costa Rica.

Isolate ID	Alias	Host	Locality
COF0209	Ca-V^a^	coffee (*Coffea arabica*)	Desamparados, San José Province, CR
PD0212	Vv-II^a^	grape (*Vitis* sp.)	San José, San José Province, CR
COF0222	Ca-I^a^	coffee (*C. arabica*)	Desamparados, San José Province, CR
COF0223	Ca-IV^a^	coffee (*C. arabica*)	Curridabat, San José Province, CR
COF0227	Ca-VII^a^	coffee (*C. arabica*)	Orosí, Cartago Province, CR
COF0245	Ca-III^a^	coffee (*C. arabica*)	Grecia, Alajuela Province, CR
COF0246	Ca-VI^a^	coffee (*C. arabica*)	Grecia, Alajuela Province, CR
COF0393	SD5	coffee (*C. arabica*)	Santo Domingo, Heredia, Costa Rica
COF0394	SD14	coffee (*C. arabica*)	Santo Domingo, Heredia, Costa Rica
COF0396	SD7	coffee (*C. arabica*)	Santo Domingo, Heredia, Costa Rica
COF0397	C12	coffee (*C. arabica*)	Curridabat, San José, Costa Rica,
COF0398	SD3	coffee (*C. arabica*)	Santo Domingo, Heredia, Costa Rica
COF0399	SD1	coffee (*C. arabica*)	Santo Domingo, Heredia, Costa Rica
COF0400	SD16	coffee (*C. arabica*)	Santo Domingo, Heredia, Costa Rica
COF0401	C17	coffee (*C. arabica*)	Curridabat, San José, Costa Rica,
COF0402	C10	coffee (*C. arabica*)	Curridabat, San José, Costa Rica,
COF0403	C6	coffee (*C. arabica*)	Curridabat, San José, Costa Rica,
COF0404	SD1	coffee (*C. arabica*)	Santo Domingo, Heredia, Costa Rica
COF0405	C11	coffee (*C. arabica*)	Curridabat, San José, Costa Rica,
COF0406	SD3	coffee (*C. arabica*)	Santo Domingo, Heredia, Costa Rica
COF0408	C13	coffee (*C. arabica*)	Curridabat, San José, Costa Rica,
PD0410	5262 grape	grape (*Vitis* sp.)	La Uruca, San José, Costa Rica
PD0411	5271 grape	grape (*Vitis* sp.)	La Uruca, San José, Costa Rica
COF0412	SD10 coffee	coffee (*C. arabica*)	Santo Domingo, Heredia, Costa Rica

a Isolates previously published in Montero-Astúa et al. [26].

### Comparison of the Temecula-1 and M23 Genomes

The Temecula-1 and M23 genomes are available at GenBank under the accession numbers AE009442.1 and CP001011.1, respectively.

Step 1: Manual alignment of the Temecula-1 and M23 genomes. Upon analysis, both genomes were found to be completely co-linear, except for a few indels.

Step 2: Identification of locally variable regions with an average genetic divergence >1%. These regions were defined by a continuous set of genetic differences between the two genomes separated by less than 458 bp, since given sequence divergence of 1%, gaps of <458 bp are expected 99% of the time.

Step 3: Classification of variable regions. First, variable regions associate with internal duplication (and hence the possibility of generating divergence through internal recombination) were identified using BLAST against the *X. fastidiosa* subsp. *fastidiosa* genomes. Second, the remaining variable regions were examined for the possibility that the divergence was due to homologous recombination with *X. fastidiosa* subsp. *multiplex*.

Step 4: Indentifying homologous recombination. The variable regions were aligned with two additional *X. fastidiosa* genomes, M12 [21], which is a fully annotated *X. fastidiosa* subsp. *multiplex* genome (GenBank CP000941.1), and Ann1 [32], which is a draft *X. fastidiosa* subsp. *sandyi* genome (GenBank AAAM00000000.3). *X. fastidiosa* subsp. *sandyi* is the closest relative of *X. fastidiosa* subsp. *fastidiosa* (see Figure 1). Regions of these two genomes corresponding to the target *X. fastidiosa* subsp. *fastidiosa* sequence were identified using BLAST and manually aligned. Alignment with Ann1 was complicated by the apparent contamination of the Ann1 database with sequence from *X. fastidiosa* subsp. *multiplex* [16]. As a result, in most cases, two alignments were produced, one identical or nearly identical to M12, which was discarded, and a second *X. fastidiosa* subsp. *sandyi* sequence that was used in the analysis. In aligned regions, a nucleotide site provided evidence for recombination of *X. fastidiosa* subsp. *multiplex* into M23 (*X. fastidiosa* subsp. *fastidiosa*) if it was identical between M12 and M23, but different from Temecula-1 and Ann1, which were themselves identical; and conversely, sites provided evidence for recombination of *X. fastidiosa* subsp. *multiplex* into Temecula-1 if the site was identical for M12 and Temecula-1, but different and identical for M23 and Ann1 (Figure 1).

### Phylogeny

To create a phylogeny of the isolates, we used a concatenation of the 8 sequenced regions for each isolate. The evolutionary model used was the maximum likelihood GTR (general time reversible) model with gamma distributed rate variation using PAUP [33]. This model was the best-fit model, as defined using FindModel (http://www.hiv.lanl.gov/content/sequence/findmodel/findmodel.html) (data not shown). Bootstrap values were based on 1,000 replicates. The sequences were visualized with BioEdit [34] and aligned manually. There was 1 indel, a 30 bp deletion shared by 12 of the 15 Costa Rican isolates. In the phylogenetic analysis this indel was weighted equivalent to 3 transversions.
